# The relationship between brain-derived neurotrophic factor and cognitive functions in alcohol-dependent patients: a preliminary study

**DOI:** 10.1186/s12991-015-0065-z

**Published:** 2015-09-23

**Authors:** Changwoo Han, Hwallip Bae, Sung-Doo Won, Sungwon Roh, Dai-Jin Kim

**Affiliations:** Department of Psychiatry, College of Medicine, Korea University, Ansan Hospital, Ansan, Korea; Department of Psychiatry, Seonam University, Myongji Hospital, Goyang, Korea; Keyo Medical Foundation, Keyo Hospital, Uiwang, Korea; Department of Mental Health Research, Seoul National Hospital, Seoul, Korea; Department of Psychiatry, Seoul St. Mary’s Hospital, The Catholic University of Korea College of Medicine, Seoul, 137-701 Korea

**Keywords:** Alcohol dependence, Cognitive function, Brain-derived neurotrophic factor

## Abstract

**Background:**

As a neurotoxic substance, alcohol can induce neurodegenesis in the brain. Alcohol-dependent patients’ cognitive functioning can be affected by chronic alcohol use. In addition, brain-derived neurotrophic factor (BDNF) is known to reflect the status of neuroadaptive changes. The purpose of this study was to investigate the relationship between cognitive functions and BDNF in alcohol-dependent patients.

**Methods:**

The subjects were 39 alcohol-dependent patients. BDNF was measured using an enzyme-linked immunosorbent assay kit. We examined clinical features and administered the Korean version of Alcohol Dependence Scale. We also used the Consortium to Establish a Registry for Alzheimer’s Disease (CERAD) to measure cognitive functioning. Then, we determined the relationships between BDNF and various parts of the CERAD.

**Results:**

The performance of alcohol-dependent patients proved stable in most parts of the CERAD. Within the different parts of the CERAD, only Trail Making Test B correlated with BDNF. Trail Making Test specifically assesses executive functions.

**Conclusions:**

BDNF might play an important role in the detection of neurocognitive function among individuals with alcohol dependence.

## Background

Alcohol use disorders are known to damage various organs or cause severe health issues [1]. Alcohol, in particular, is an agent of diverse neuronal damages, including an increase in apoptosis, a decrease in the proliferation of nerve cells, and impairment of neural networks [2, 3]. As for the major mechanism of alcohol-induced neurodegeneration, early research has highlighted that chronic alcohol consumption causes excitotoxicity, thus activating glutamatergic transmission and ultimately resulting in neuronal damage via intracellular signal pathways [4]. Neuronal damage has been shown to inhibit neurogenesis and to decrease dendritic networks of Purkinje neurons in the brain [5]. Moreover, alcohol-induced modification of the cranial neural structure has been observed in quite a few parts of the brain (e.g., the corticolimbic area, hippocampus, and the entorhinal cortex) [6, 7]. Importantly, since the hippocampus is involved in memory or cognitive functioning in the brain, long-term alcohol consumption could damage the hippocampus, causing cognitive impairment [8].

As polypeptide compounds, neurotrophins are mostly distributed in the nervous system, and are involved in neuronal activation, differentiation, and survival in the brain [9, 10]. As a representative neurotrophin, brain-derived neurotrophic factor (BDNF) is known to be involved in neuronal growth, differentiation, synaptic connection, modification, and survival [11]. Previous studies have found associations between BDNF and depression, schizophrenia, and other mental illnesses [12]. Notably, as compared to a control group comprising subjects without depression, patients with depression show lower BDNF levels [13]. In patients with depression, the volume of the limbic system, including the hippocampus, has been found to decrease; in view of this, it has been reported that depression is related to a decline in neurogenesis and several neurofactors [14]. Thus, the decreasing BDNF in patients with depression seems to reflect such types of neurodegeneration. Since BDNF is involved in neuronal survival and resistance to degeneration, BDNF is considered an indicator of alcohol-induced neurotoxicity. Previous research has found low BDNF level in alcohol-dependent patients [15]. Alcohol-induced blockage of *N*-methyl-d-aspartate (NMDA) receptors decreased the expression of BDNF, which in turn increased neurotoxicity [16].

One of the salient clinical symptoms of alcohol dependence that is associated with alcohol-induced injury of cranial nerves is the decline in cognitive functions. It is a well-known fact that chronic alcohol consumption may temporarily damage cognitive functions and cause permanent structural impairment of brain [17]. Alcohol-dependent patients reportedly show a decline in learning and memory, among other cognitive functions [18], and display psychomotor retardation, circumstantiality, decreased attention, and disorders of orientation [19]. Moreover, chronic alcohol consumption may bring about a decline in executive functions related to attention, problem-solving abilities, and self-control [20]. In turn, the impairment of executive functions leads to deterioration in patients’ social adjustment and functional rehabilitation, serving as a primary cause of a poor prognosis [21].

BDNF has been found to mirror the cognitive decline accompanying mental illnesses. Among patients with schizophrenia, a mental illness that is most likely to be accompanied by a decline in cognitive functions, an association was found between BDNF and verbal working memory and negative symptoms [22]. Among patients with depression, a correlation was found between cognitive functions and BDNF [23]. BDNF is regarded as a biological marker reflecting a decline in memory and general cognition during the aging process [24].

However, the relationship between alcohol-dependent patients’ cognitive functions and BDNF has never been investigated. In an experiment on mice, long-term alcohol intake decreased hippocampal BDNF gene expression [25]. This finding suggests that, in terms of hippocampal physiology, BDNF is involved in alcohol-induced neurodegeneration. Therefore, BDNF is a viable biomarker of the cognitive functions of alcohol-dependent patients.

Either neurocognitive function tests such as the Wechsler Memory Scale and the Wisconsin Card Sorting Test, or the categorical subsets of the Halstead–Reitan battery, are used to measure diverse cognitive functions of alcohol-dependent patients [26, 27]. In addition, a study used the Trail Making Test, which involves alternate and sequential connection of numbers and alphabet letters, to measure cognitive flexibility [28]. Another study used the Consortium to Establish a Registry for Alzheimer’s Disease (CERAD) to examine the cognitive functions of patients with alcohol problems [29].

In order to establish the nature of the relationship between cognitive functions and BDNF among alcohol-dependent patients, this study assessed cognitive functions among this sub-group and their BDNF levels. Subsequently, the correlation between the two was determined, thus shedding light on the legitimacy of BDNF as a nerve factor indicating alcohol-dependent patients’ cognitive functions.

## Methods

### Subjects

Thirty-nine inpatients in a psychiatric hospital with a specialized alcohol treatment center located in Gyeonggi-do participated in this study. These patients had been diagnosed with alcohol dependence, based on the DSM-IV criteria. Their ages ranged from 37 to 62 years. Those with a history of severe cranial head injury or who had comorbid other mental illnesses were excluded from the study. In addition, those with addictions or a history of abusing other substances were excluded from the study. Subjects were confirmed to have abstained from alcohol for at least a week. Because previous studies have found differences in some growth factors among individuals with alcoholic withdrawal symptoms [30, 31]. Those with withdrawal symptoms were excluded from the experimental group.

Table 1 shows the subjects’ demographic and clinical characteristics. This study was conducted with the approval of Seoul St. Mary’s Hospital’s Clinical Trial Review Board (C11QISI0569). The author informed the subjects about the objective of this study. All subjects were male, voluntarily participated in the study, and signed the consent form.

**Table 1:** Demographic characteristics and alcohol-related data

	Mean ± SD (range)
Age	47.98 ± 6.38 (25–71)
Education (years)	10 ± 3.64 (6–16)
Onset age of drinking	22.35 ± 8.51 (15–53)
K-ADS	22.98 ± 12.27 (0–46)

*K-ADS* Korean version of Alcohol Dependence Scale

### Clinical measures

Sociodemographic data, such as age, education, and age of initial alcohol use were obtained from a questionnaire survey. In order to assess the severity of alcohol dependence, the Korean version of the Alcohol Dependence Scale (ADK-K) was administered; the scale examines obsessive drinking, behavioral control over drinking, and withdrawal symptoms. It consists of 25 items developed by Skinner and Allen through factor analysis. ADS-K is the Korean version of Alcohol Dependence Scale, which was standardized by Lee et al. for the Korean context [32]. The Korean version of the CERAD (CERAD-K) was used to evaluate cognitive functions. This 9-item neuropsychological test evaluates language, memory, and composition and performance abilities [33]. The CERAD-K is the Korean edition of the neuropsychological battery developed by the CERAD, which was established in the US [34].

### Blood analysis

A nurse collected blood samples from subjects at 10–11 a.m. All the subjects had breakfast at 8 a.m. and rested or attended the ward information program, without engaging in exercise or activities requiring high output of physical energy prior to the collection of blood samples. The blood samples (10 ml each) were placed in an EDTA-coated tube and immediately centrifuged. Then, plasma samples were collected and kept at −80 °C until analysis.

The Human Brain-Derived Protein Multiplex Immunoassay Kit was used to measure BDNF. All products were from Millipore (Millipore Korea, South Korea). All procedures were conducted according to standard guidelines provided by the manufacturer. Each plate’s concentration was corrected by means of its standard curve’s dilution factor.

### Statistical analysis

Descriptive analyses were performed on demographic variables, including age, education, and age of initial alcohol use, as well as scores on the ADS-K items. Subjects were divided into two groups, depending on family history of alcohol dependence in parents, parents’ siblings, and grandparents (i.e., first- or second-degree relative). Then, an independent-samples *t* test was performed to verify the difference in BDNF between the two groups.

A correlation analysis was conducted to determine the relationship between BDNF and various neurocognitive function measures included in the CERAD-K. Windows SPSS (Statistical Package for Social Sciences) 20.0 was used for statistical analyses, with the significance threshold of 0.05 applied to all analyses.

## Results

1. Demographic variables and characteristics of alcohol dependence problems
  Table 1 presents data on age, education, initial alcohol use, frequency of drinking, and scores on the ADS-K. The mean age was 48 years and mean education duration was 10 years. The mean age of initial alcohol use was 22 years, and mean ADS-K score was 23.
2. Relationship between alcohol dependence and neurocognitive functions
  With regard to MMSE-K total scores, six patients (15 %) scored below 23, which indicated a decline in overall cognitive functions (Table 2).

**Table 2:** Results of the CERAD-K

CERAD-K	Mean ± SD
Verbal fluency	15.15 ± 3.62
Word list memory	18.15 ± 4.00
Word list recall	6.45 ± 2.07
Word list recognition	9.30 ± 0.99
Constructional praxis	10.40 ± 1.17
Constructional recall	8.50 ± 2.84
Boston naming test	12.18 ± 1.92
MMSE-K	26.67 ± 3.16
TMT-A	52.62 ± 25.38
TMT-B	192.20 ± 9216

*CERAD-K* Korean version of the Consortium to Establish a Registry for Alzheimer’s Disease, *TMT* Trail Making Test
3. Differences in BDNF levels with respect to family history of alcohol dependence
  BDNF levels were significantly lower in the group with a family history of alcohol dependence than that without such a family history (*t* = −2.596, *p* = 0.013) (Table 3).

**Table 3:** Differences in serum BDNF levels of alcohol-dependent patients with/without family history (*n* = 39)

	A without FH	A with FH	*t*	*p*
	*n* = 17	*n* = 22		
BDNF (pg/ml)	3474.17	2636.90	−2.596	0.013*

A without FH: alcohol-dependent patients without family history of alcohol dependence

A with FH: alcohol-dependent patients with family history of alcohol dependence

*BDNF* brain-derived neurotrophic factor

* *p* < 0.05
4. Relationship between BDNF and neurocognitive functions
  The relationship between BDNF and ADS-K scores was not significant. Moreover, with the exception of the correlation found between Part B of the Trail Making Test and BDNF (*r* = 0.337, *p* < 0.05), no significant relationships were found between most tests of the CERAD-K battery and BDNF (Table 4).

**Table 4:** Correlations between BDNF, TMT-A, and TMT-B

	BDNF	TMT-A	TMT-B
BDNF	1		
TMT-A	0.153	1	
TMT-B	0.337*	0.668**	1

*BDNF* brain-derived neurotrophic factor, *TMT* Trail Making Test

* *p* < 0.05

** *p* < 0.01

## Discussion

The present study was intended to investigate the relationship between alcohol-dependent patients’ cognitive functions and the neurotrophin BDNF. Patients with a family history of alcohol dependence showed lower BDNF levels than those without such a family history. This finding is consistent with previous research [15]. Accordingly, alcohol-dependent patients with a family history of alcohol dependence are more likely to develop neurodegenesis than patients without such a family history.

Among alcohol-dependent patients, most of the scores obtained on the CERAD tests did not show any explicit correlation with BDNF levels. Nevertheless, a correlation was found between BDNF and Part B of the Trail Making Test, which measures performance abilities. Given that the relationship between alcohol dependence and neurotrophins has hardly been investigated, the present finding that BDNF is likely to reflect alcohol-dependent patients’ cognitive functions is specific.

The Trail Making Test is primarily used to measure executive functions. Since the test includes measurement of motor components and complex visual scanning, it can be used as a visual–motor tracking test. In addition, the Trail Making Test is associated with sustained attention and psychomotor speed [35]. As a rule, compared to the relatively less complex Part A of the Trail Making Test, Part B of the same test involves the alternate connection of numbers and letters, thus taking longer to complete because it requires complex cognitive processes drawing on faster motor speed and visual scanning, and is affected by acquired learning abilities such as education levels [36]. Thus, based on the present findings, BDNF might be indicative of alcohol-dependent patients’ executive functions.

Apart from alcohol-dependent patients, a study on the relationship between depressed patients’ cognitive functions and BDNF showed a correlation between Part B of the Trail Making Test, among other neurocognitive tests, and cognitive functions [23]. This study confirmed a decline in the cognitive functions of patients with depression, as compared to a control group of subjects without depression. The study also found a negative correlation in the linearity check between Part B of the Trail Making Test and BDNF.

There are varying reports on the relationship between mental illness and BDNF. However, the precise nature of this relationship has not been determined. Moreover, findings vary with regard to serum or plasma measurement [37]. Unlike depression, which reflects chronic neurodegenerative conditions, physiological states are highly likely to change in line with alcohol intake, including the duration of abstinence or amount of alcohol consumption in instances of dependence. Notably, a range of neurological changes could occur during the hyper-excitable response state (e.g., the alcohol withdrawal state). Research on alcohol withdrawal in relation to BDNF has shown that, among members of the abstinence group, rather than the non-abstinence group, BDNF levels increased to a greater extent, following the commencement of the withdrawal state [31]. Another study on alcohol-dependent patients reported that BDNF levels increased following alcohol detoxification [38]. This finding seems attributable to a BDNF-induced neuroadaptive process.

Similar to nerve growth factor, BDNF is converted from probrain-derived neurotrophic factor (proBDNF). Although the process of proBDNF is still unclear, proBDNF, like BDNF, may serve as neuronal signals at nerve terminals. BDNF promote the proliferation of the active nerve terminals, whereas proBDNF demote the less active one [39]. ProBDNF suppressed synaptic transmission and induced hippocampal depression [40, 41]. A previous study showed that proBDNF was correlated with the depression [42, 43]. In these studies, the role of the proBDNF in signaling pathways of the dysregulation in depression should be suggested as biomarkers for the major depression. However, another study showed that serum levels of mature BDNF in depressive patients were significantly lower than in normal controls, but there was no difference in the serum levels of proBDNF [44]. In our study, we could not involve the kit for detecting proBDNF. Therefore, our measurement could not distinguish between BDNF and proBDNF.

In the present study, the patients were hospitalized in an alcohol treatment ward for more than a week, thus abstaining from alcohol for a long period. Therefore, the positive correlation found between BDNF levels and the Trail Making Test seems to reflect restoration of the BDNF following alcohol-induced neurodegeneration. Or the influence of the level of proBDNF could not be excluded absolutely. Therefore, further studies are needed to identify the role of proBDNF in alcohol dependence.

In most instances, the cognitive deficits of alcohol-dependent patients are known to improve with continued abstinence, as compared to persistence of the withdrawal or intoxicated state [45]. However, some patients remain cognitively dysfunctional despite a considerable time lapse, which affects patient outcome and adaptation to life after treatment [19]. In particular, alcohol-dependent patients display learning disorders, deficits relating to abstract reasoning and performance of complex tasks, and slow recovery in relation to these even when they abstain from alcohol [46]. Therefore, further studies should examine the relationships between alcohol dependence, neurotrophins, and the mechanism of neurotrophins in the cognitive function deficits of alcohol-dependent patients.

The present study sought to explore the relationship between alcohol-dependent patients’ cognitive functions and BDNF; however, some limitations were identified.

First, this study was a lack of comparison between alcohol-dependent patients and the control group, and the number of experimental subjects was limited. Moreover, all the subjects were male patients, which meant that no inferences could be made about female alcohol-dependent patients. In order to clarify whether BDNF is an indicator of alcohol-dependent patients’ cognitive functions, future studies should use large sample sizes that would enable a comparison of alcohol-dependent groups with non-alcohol dependent control groups.

Second, although the patients studied here had abstained from alcohol for more than a week and showed no withdrawal symptoms, abstinence was not prolonged. Therefore, the patients’ physiological states following alcohol detoxication may have varied. In order to observe differences in BDNF levels according to abstinence, further studies should compare patients’ subsequent physiological states, taking into account differences in the duration of abstinence.

Third, we could not include proBDNF. As a precursor of BDNF, proBDNF can be useful to identify the mechanism of the neurodegeneration in the psychiatric diseases. There is a need to examine proBDNF in follow-up studies.

Lastly, the present study used the CERAD to measure cognitive functioning. However, the CERAD primarily measures neurocognitive degeneration and is generally administered to patients with diseases that accompany the aging process. Therefore, the CERAD is not suitable for measuring cognitive functions in alcohol-dependent patients. Although in the current study, the CERAD was used to assess overall cognitive functions and performance abilities, further studies should employ instruments that are capable of testing more complex performance abilities.

## Conclusions

This study established a relationship between the neurotrophin BDNF and Part B of the Trail Making Test, which reflects alcohol-dependent patients’ performance abilities among other cognitive functions. The findings suggest that BDNF might serve as a biomarker mirroring neuroregenesis among patients with alcohol problems and as a protective factor against alcohol-induced neurodegeneration.

Further studies should investigate the correlation between cognitive functioning and BDNF actions in reference to the pathology of declining cognitive functions in alcohol-dependent patients.
